# Episodic future thinking together with observational learning benefits prospective memory in high‐functioning Korsakoff’s syndrome patients

**DOI:** 10.1111/bjc.12251

**Published:** 2020-05-18

**Authors:** Beth Lloyd, Erik Oudman, Mareike Altgassen, Serge J. W. Walvoort, Roy P. C. Kessels, Albert Postma

**Affiliations:** ^1^ Helmholtz Institute Experimental Psychology Utrecht University The Netherlands; ^2^ Slingedael Korsakoff Center Rotterdam The Netherlands; ^3^ Donders Institute for Brain Cognition and Behaviour Radboud University Nijmegen The Netherlands; ^4^ Department of Psychology Johannes Gutenberg-University Mainz Germany; ^5^ Centre of Excellence for Korsakoff and Alcohol‐Related Cognitive Disorders Vincent van Gogh Institute for Psychiatry Venray The Netherlands; ^6^ Department of Medical Psychology Radboud University Medical Center Nijmegen The Netherlands

**Keywords:** alcoholics, future event stimulation, future thinking, Korsakoff’s syndrome, observational learning, prospective memory

## Abstract

**Objective:**

Patients with Korsakoff’s syndrome (KS) have difficulty carrying out tasks which rely on prospective memory (PM). Since remembering to carry out an action in the future is crucial for living independently, it is of primary interest to develop strategies that improve PM performance in KS patients.

**Design:**

The study employed a computer categorization task as an ongoing activity into which a PM task was embedded. We included episodic future thinking (EFT) and observational learning (Experiment 2) to boost PM.

**Methods:**

Experiment 1 evaluated the efficacy of EFT following written PM task instructions in ten KS patients. Due to floor‐level PM performance in Experiment 1, Experiment 2 included an instructional video demonstrating the PM intention. In Experiment 2, twenty‐six KS patients performed both conditions (EFT and no‐EFT) at least 1 week apart, while twelve controls with alcohol use disorder without KS performed the no‐EFT condition. In Experiment 2, the PM instructions were also shown through video (observational learning component). Mild cognitive impairment was assessed in a short test battery.

**Results:**

Experiment 1 showed overall floor performance in both conditions. Experiment 2 showed that KS patients performed PM tasks less accurately than the control group in the no‐EFT condition. In Experiment 2, where the observational learning component was included, EFT improved PM performance in KS patients. This effect was driven by a sub‐group of high‐functioning KS patients.

**Conclusions:**

This study showed the value of an observational learning component together with EFT in improving PM performance, in relatively high‐functioning KS patients.

**Practitioner points:**

- KS patients performed the PM task less accurately than non‐KS controls with alcohol use disorder, confirming PM impairment in this patient population.
- Controls with alcohol use disorder performed the PM task at ceiling level.
- Showing an instructional video demonstrating the PM intention improved PM performance and later recall of PM task instructions in KS patients.
- Episodic future thinking strategy improved PM performance in KS patients with relatively intact cognitive functioning.

Korsakoff’s syndrome (KS) is a neuropsychiatric disorder often caused by the combination of chronic and excessive alcohol consumption and severe malnutrition. Depleted thiamine (vitamin B1) is regarded as the major aetiological factor resulting in KS (Arts, Walvoort, & Kessels, 2017). KS is characterized by severe cognitive deficits as a result of lesions in the diencephalon; in particular, the mammillary bodies and the thalamus are affected (Arts *et al.*, 2017; Fama, Pitel, & Sullivan, 2012). These cognitive deficits predominantly impair patients’ episodic memory and executive functioning (Brion, Pitel, Beaunieux, & Maurage, 2014; Fama, Marsh, & Sullivan, 2004).

An aspect of episodic memory that is particularly impaired in KS concerns the learning of new information, leading to anterograde episodic memory deficits (Kopelman, 2002). Episodic memory deficits in KS have been reported when performing a variety of tasks, such as spatial memory, memory for autobiographical events, as well as word‐list learning (Butters, Tarlow, Cermak, & Sax, 1976; Kessels, Postma, Wester, & De Haan, 2000; Postma, Morel, Slot, Oudman, & Kessels, 2018; Postma, Van Asselen, Keuper, Wester, & Kessels, 2006). In addition to episodic memory deficits, overall impairments in executive functioning are often observed in these patients (Moerman‐Van Den Brink *et al.*, 2019). Executive functions allow us to plan, control, adapt, select and monitor behaviour in response to novel situations (Alvarez & Emory, 2006; Miyake *et al.*, 2000). KS patients perform poorly on a wide range of tests measuring executive abilities, such as planning, goal‐oriented actions, inhibitory control, working memory and flexibility (Brand *et al.*, 2003, 2005; Fujiwara, Brand, Borsutzky, Steingass, & Markowitsch, 2008; Joyce & Robbins, 1991; Pitel *et al.*, 2008).

Interestingly, episodic memory and executive functioning support a common mechanism, a process known as prospective memory (PM; Einstein & McDaniel, 1990). Successful PM enables one to anticipate a future intention, such as sending an email to your boss and then to plan, coordinate and execute the intention at the appropriate moment in the future (Gollwitzer, 1999). Significant examples of PM tasks include remembering to take medication at certain points in the day or attending hospital appointments on time. In such cases, poor PM could result in life changing consequences. It is therefore surprising that PM has not been extensively studied in KS.

Prospective memory tasks typically include two main components, a ‘what’ and a ‘when’ component. These commonly depend on episodic memory and executive functions. The content of the intention must be recalled, that is the ‘what’ component, which heavily relies on episodic memory (Einstein & McDaniel, 1990; Prigatano & Klonoff, 1998). Executive processes are needed to monitor the environment for cues, to inhibit and switch tasks in order to perform the prior intention, mechanisms which come into play during the ‘when’ component of a PM task. These two processes (episodic memory and executive functions) are severely impaired in KS, and previous studies have already demonstrated that KS patients perform considerably poor on PM tasks (Altgassen, Ariese, Wester, & Kessels, 2016; Brunfaut, Vanoverberghe, & D’Ydewalle, 2000).

Few studies have investigated possible strategies for improving PM task performance in KS. Brunfaut *et al. *(2000) showed that KS patients have severely compromised PM functioning. In their task, participants were asked to count the number of letters in each presented noun. In target words, for all animals, participants had to press the red coloured space bar. KS patients had almost floor performance. In a second task, participants were asked to explain the meaning of presented words and to press the space bar for animals. In this experiment, KS patients performed relatively well, possibly because the PM task and ongoing task were more comparable. Altgassen *et al. *(2016) found that increased saliency led to improved PM performance in KS patients. In their task, patients carried out an ongoing computer task involving categorizing objects and were required to push the space bar whenever a particular object was presented (e.g., cat). In a high‐salience condition (where a red outline was presented around the PM cue) compared to a low‐salience condition (no outline), KS patients performed significantly better. This study provides evidence that high‐salience cues can aid KS patients in overcoming their PM deficit. A recent case study reported that an external aid such as a smartwatch can potentially support PM functioning in KS (Lloyd, Oudman, Altgassen, & Postma, 2019). The foregoing interventions yielded their effects by offering external support during the later stages of PM (i.e., the retrieval phase). However, daily routines should not only have to depend on external prompts and cues, but ideally may also benefit from enhanced self‐generated monitoring. For this to occur, early stages of PM (i.e., intention encoding) must be stimulated. As far as we know, there is no research yet on the question of whether KS patients can indeed be trained at the encoding stage, to better keep track of PM assignments.

In this light, the current study investigated the effectiveness of a cognitive approach in aiding the first stage of PM processes, namely planning when and how the intention will be performed, in KS patients. Episodic future thinking (EFT) is the capacity to simulate possible future events by drawing on elements of past experiences (Atance & O’Neill, 2001; Schacter & Addis, 2007; Schacter, Addis, & Buckner, 2007; Tulving, 1983). The idea is that when receiving a PM assignment, the person in question mentally ‘pre‐experiences’ the future. For example, imagine that you are preparing to take your driving test, you lay out the possibilities of which manoeuvres you may be asked, and one by one you imagine yourself carrying these out. You draw on aspects of the scenario from your previous driving lessons and simulate the scene, this time with an examiner by your side. This fictional (yet relatable) scenario is an adequate example of what is termed EFT. The prospect that EFT could prove beneficial for KS patients in PM tasks comes from four previous studies, which applied this strategy in non‐patient groups. Altgassen *et al. *(2015) found that both cognitively unimpaired younger and older adults benefited from an instruction that required them to simulate themselves executing the PM tasks involved in the Dresden Breakfast Task, where participants are asked to carry out time and event‐based tasks to prepare breakfast for four people. Two studies also investigated the effect of EFT on PM tasks in healthy individuals exposed to acute alcohol consumption, both reported significant improvements during the EFT condition (Leitz, Morgan, Bisby, Rendell, & Curran, 2009; Paraskevaides *et al.*, 2010a). Lastly, Mioni *et al. *(2017) found that an EFT strategy resulted in fewer PM deficits in traumatic brain injury patients. Considering these positive results, and the value of finding an effective strategy for improving PM in KS, we designed this study to measure the effect of EFT on PM performance in KS patients.

To test this hypothesis, we conducted two experiments. We used a mixed‐subject design to compare the performance of two groups (KS patients and patients with alcohol use disorder [AUD]) on a computerized PM task. KS patients performed the task once with no strategy (no‐EFT condition), and once after carrying out EFT (EFT condition). In Experiment 1, the PM task was presented with on‐screen worded instructions (no pictures or animation). However, patients with KS were unable to perform this version of the PM task. We therefore adapted the experimental design in Experiment 2. Experiment 2 included two manipulations compared to Experiment 1: first, an observational learning manipulation (i.e., the use of an instructional video concretely displaying the PM task), which was carried out in all conditions. Since KS patients have deficits in abstract reasoning and verbal episodic memory, we reasoned that observational learning may help patients learn the PM task, since observational learning does not overly rely on abstract reasoning or verbal memory, and has improved learning in other patient groups (Shohamy *et al.*, 2004; Van Tilborg, Kessels, & Hulstijn, 2011); and second, the EFT manipulation, which was carried out in one of the two sessions. Finally, since there is large variability in cognitive functioning across KS patients, we also applied the Montreal Cognitive Assessment (MoCA; Nasreddine *et al.*, 2005) in both experiments, to account for the ‘general’ level of cognitive impairment. In line with previous studies, we predicted that KS patients would perform better on the PM task after applying EFT compared to when no strategy is instructed.

## Experiment 1

### Method

#### Participants

Ten KS patients participated in this experiment. These patients were recruited from a clinic in the Netherlands. Data from nine KS patients (seven men) were included. One patient was excluded due to illness over the testing period. All KS patients met the diagnostic criteria of the DSM‐5 for major neurocognitive disorder due to alcohol (Nuckols & Nuckols, 2013) and the criteria for KS as described by Kopelman (2002). All patients had been detoxified for at least 2 months and were in the chronic, amnesic state of KS; none were in the state of Wernicke psychosis during the time of testing.

The MoCA (Nasreddine *et al.*, 2005) was conducted on all patients. The education level of the patients was determined using seven categories in accordance with the Dutch educational system, one being the lowest (< primary school) and seven being the highest (> academic degree; Verhage, 1964). This study was approved by the ethics board of the University and by the institutional review board of the clinic. Informed consent was obtained in all patients. Table 1 shows demographic variables and MoCA scores for all nine patients.

**Table 1:** Demographic variables and MoCA scores of KS patients from Experiment 1

	Korsakoff patients (*N* = 9)
Sex distribution (m:f)	7:2
Age (mean + *SD*)	63.1 (7.2)
Education level (mean + *SD*)	4.8 (1.2)
MoCA score (mean + *SD*) (Max score: 30, cut‐off score: 26)	18.2 (3.3)

#### Prospective memory task

**Figure 1:**
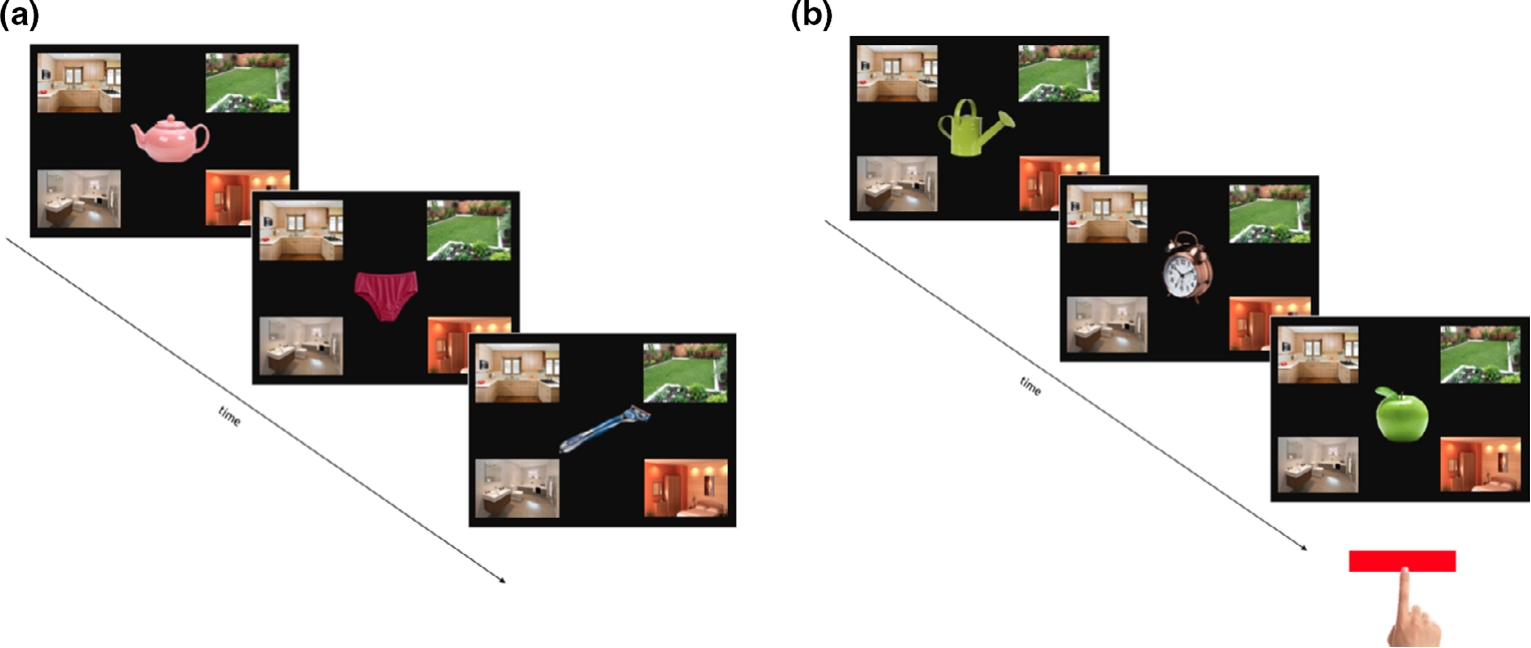
Example of the task paradigm. An overview of the ongoing categorization task is illustrated in (a) and the prospective memory task with the cue stimulus and cue responses is presented in (b). [Colour figure can be viewed at wileyonlinelibrary.com]

A computerized object‐room categorization task was used in which patients had to indicate in which room the trial object typically belonged. The task was presented on a touch‐sensitive screen and designed with the software package Psychopy2 (Peirce, 2007). The rooms (kitchen, bathroom, bedroom, garden) remained on the screen situated in each corner, with the household objects (e.g., milk, plant, pillow) presented in the centre of the screen (see Figure 1). To select an appropriate room, patients pressed the picture of the room with their finger. Patients first read the ongoing task instructions; they then completed a practice block, the practice block ended after patients responded correctly to ten consecutive trials. Feedback and repeated instructions were provided during the practice block as necessary. Once the task was learned, patients completed block one which consisted of 20 trials with an ITI of 2.5s, no feedback was given during this block.

After block one, the PM task instruction was introduced. The second block required patients to complete two tasks simultaneously, the previously learned categorization task and an added event‐based PM task (see Figure 1b). The PM task required patients to remember to respond differently to one type of object, this was known as the ‘PM cue’. Two PM cues were used, an apple and a toothbrush, these were counterbalanced over the two conditions. The patients were instructed to push the space bar when the PM cue appeared, instead of placing it into its corresponding room (i.e., apple into the kitchen and toothbrush into the bathroom). The space bar was red or orange to indicate which button to push (counterbalanced over conditions). After the PM task instruction had been read and understood, either the EFT manipulation took place (EFT condition) or a distractor task followed (no‐EFT condition).

The second task block (with the added PM task) comprised of 45 trials, seven of which were PM cues. At the end of the second block, all patients were asked to recall the instructions for the first and the second block, including the PM task instruction. Expected answers included what they had to do in the categorization task and what the PM task instruction was. Prompts were provided if the patient could not produce an answer. This recall question was to ensure the PM task was properly comprehended and successfully encoded.

#### Episodic future thinking instruction

The EFT instruction was counterbalanced over conditions. The instruction followed a two‐step procedure. Patients were first asked to practise future thinking with an example unrelated to the task. Training scenario: imagine calling a friend/family member before going to bed. Patients were asked to close their eyes for 20s and imagine how this event would take place in as detailed a manner as possible. This component took around 3 min. Following the training component, patients were asked to apply this strategy to the PM task instruction. They were asked to vividly imagine themselves completing the categorization task and upon seeing the PM cue, to respond by pushing the space bar. They closed their eyes and did this for 20s; they were prompted to include as much detail as possible. In total, the EFT procedure lasted around 5 min.

#### Distractor task

The distractor task took place directly after presenting the PM task instructions in the no‐EFT condition or after the EFT component in the EFT condition. Patients were directed away from the computer and onto the pencil and paper MoCA task. The distractor task included the ‘visuospatial/executive’ and the ‘naming’ sections of the MoCA test. This lasted maximum 5 min. Two versions of the MoCA were used for the distractor task (version 7.1 and 7.3), in order for patients to complete different task content in each condition.

#### Procedure

The within‐subject component (EFT and no‐EFT) was counterbalanced over the two conditions and took place at least 1 week apart. In the EFT condition, the EFT instruction was given, in the no‐EFT condition, the EFT instruction was not given. Dependent variables included the correct PM cue response (raw), ongoing task accuracy (%) and PM task instruction recall rate (proportion).

### Results and discussion

#### Computer categorization task

We compared the performance on block one (ongoing task only) and block two (ongoing task with PM task). There was no difference in performance on the ongoing task over these blocks [*t*(8) = 1.52, *p* = .168]. Overall, accuracy on the categorization task was high (block 1: 95.8%, block 2: 88.7%).

#### Prospective memory task accuracy

A quantitative analysis of PM task accuracy was not possible. This was due to observing floor performance in both the EFT and no‐EFT conditions (*median = *0, correct responses to the PM cue). Overall, one patient correctly responded to one PM cue; thus, no statistics were applied. Furthermore, a qualitative analysis of PM retention informed us that only 16.6% of the time the PM task instruction was successfully recalled at the end of the task. This was calculated by taking the proportion of correctly recalled instructions from all individuals over both conditions (a total of 18 observations over nine individuals).

Experiment 1 aimed to assess the beneficial effect of EFT on PM performance in KS patients. High accuracy of the patients’ performance on the computer categorization task suggests that patients did not find this task to be too difficult. The PM task instruction, however, was not successfully encoded in most cases, evident by the responses given to the follow‐up question, asking if the individuals could recall the PM task instructions upon task completion. Few patients recalled the PM task instructions, even when provided with prompts, such as ‘do you remember any instructions about an apple?’. This encoding stage is crucial in the PM process. This outcome, therefore, led us to make adaptations in the experimental design resulting in Experiment 2. In Experiment 2, the design of the task was altered from Experiment 1 to include an observational learning manipulation in the form of an instructional video. This video was presented to all participants in both conditions in Experiment 2. As well as an altered design, the second experiment included a control group of AUD patients with no KS. We expected that this group would outperform the KS patients during the no‐EFT condition and that KS patients would perform similarly to the control group when they apply EFT.

## Experiment 2

### Method

#### Participants

Experiment 2 included two groups, a KS patient group and a control group of patients with AUD. Patients were recruited from two clinics in the Netherlands. The KS group consisted of 26 KS patients (15 men) and the control group of 12 age‐and education‐matched patients with AUD (seven men). All KS patients met the diagnostic criteria of the DSM‐5 for major neurocognitive disorder due to alcohol (Nuckols & Nuckols, 2013) and the criteria for KS as described by Kopelman (2002). All patients were detoxified for at least 2 months and all were in the chronic, amnesic state of KS, none were in the state of Wernicke psychosis during the time of testing.

The MoCA (Nasreddine *et al.*, 2005) was conducted on all patients. The education levels for both patient and control groups were determined using seven categories, one being the lowest (< primary school) and seven being the highest (> academic degree; Verhage, 1964). This study was approved by the ethics board of the University and of the two clinics. Informed consent was obtained in all patients. Table 2 contains the demographic variables and MoCA scores for all patients.

**Table 2:** Demographic variables and MoCA scores of KS patients and alcoholic controls from Experiment 2

	Control group (*N* = 12)	Korsakoff patients (*N* = 26)
Sex distribution (m:f)	7:5	15:11
Age (mean + *SD*)	62.2 (7.1)	59.9 (6.9)
Education level (mean + *SD*)	4.2 (1.4)	4.4 (1.2)
MoCA score (mean + *SD*) (Max score: 30, KS patient cut‐off score: 26)	23.5 (4.3)	19.5 (4.6)

#### Procedure and analysis

The task was the same as in Experiment 1. The procedure differed only in the way the PM task instruction was communicated. Following the on‐screen text instructions of the PM task, a video of a person completing the PM intention was presented to all patients. The video was filmed in a testing room the same or similar to the one that patients were tested in. It was filmed from the view of the patient with no audio. This instructional video component was presented to both groups (patients and controls), in both conditions (EFT and no‐EFT).

The within‐subject component (EFT and no‐EFT) was counterbalanced over the two conditions and took place at least 1 week apart. In the EFT condition, the EFT instruction was given, in the no‐EFT condition, the EFT instruction was not given. KS patients took part in both conditions, and the control group only took part in the no‐EFT condition due to expected ceiling effects.

To compare performance on the computer categorization task (placing objects into their corresponding rooms), the average scores of KS patients from both conditions were calculated; these were compared to the scores of the control group. Performance on the computer categorization task in block one and block two (with the added PM task) using a 2 (group: KS patient vs. controls) × 2 (block: single task vs. dual task) mixed ANOVA.

The PM score was determined by computing the number of binary correct/incorrect responses to the PM cue (maximum score = 7). PM score was then compared between groups (KS patient and control group) in the no‐EFT condition using a Mann–Whitney *U* test.

To investigate how the EFT instruction impacted PM performance within KS patients, we fitted generalized linear mixed models (GLMMs) with a logic link function using the lme4 R package (Bates, Mächler, Bolker, & Walker, 2015). In order to maximize the generalizability of these models, we treated individuals as random effects for both the intercept and all slopes of the fixed effects included in the model. Fixed effects included the condition (no‐EFT or EFT) and Order (Order 1 = no‐EFT then EFT, Order 2 = EFT then no‐EFT). The outcome variable was PM performance, which was treated as count responses (possible number of correct responses from 0 to 7). To investigate how individual levels of cognitive functioning impacts the effect of EFT on PM, individual MoCA scores were included as a fixed effect.

### Results and discussion

#### Computer categorization task: Korsakoff’s syndrome patients versus alcoholic controls

One patient from the control group scored more than two standard deviations below the mean in their accuracy on the ongoing task; therefore, their data were excluded from analysis. Both groups performed with high accuracy on the computer categorization task in block one [*M = *95.0, *SD = *5.06] (KS patients), [*M = *96.4, *SD = *5.0] (control group) and block two [*M = *93.2, *SD = *6.5] (KS patients), [*M = *97.4, *SD = *3.1] (control group). There was no significant main effect of carrying out the PM task in combination with the categorization task [*F* (1, 36) = 0.133, *p* = .717], and the groups did not differ in terms of accuracy on the categorization task [*F*(1, 36) = 2.54, *p* = .119]. The interaction of block two by group was also not significant [*F*(1, 36) = 1.77, *p* = .192]. Overall, these results suggest that patients and controls performed equally well on the computer categorization task across both blocks.

#### Prospective memory task with no‐EFT: Korsakoff’s syndrome patients versus alcoholic controls

PM performance in the no‐EFT condition between groups differed significantly: *U*(37) = 35.000, *p* < .001. KS patients performed significantly worse in the PM task (KS patients *median = *0, control group *median = *7). This finding is further evidence for the significant memory deficits KS patients.

#### Effect of EFT on prospective memory task: Korsakoff’s syndrome patients

For descriptive statistics of PM performance as a function of condition (no‐EFT and EFT) and task Order (no‐EFT then EFT, EFT then no‐EFT), see Table 3. First, to test the effect of EFT on PM performance, we treated Instruction (no‐EFT or EFT) as the sole independent variable to predict PM performance. Estimates of the model revealed a significant effect of Instruction on PM score [*z* = 2.37, *p* < .05, β = 0.54]. Next, to check for order effects, we added Order (no‐EFT then EFT, EFT then no‐EFT) as an additional predictor. The additional variable showed no significant relationship with PM performance [*z* = 0.17, *p* < .862, β = 0.24], nor did we find an interaction effect between Order and Instruction in predicting PM performance [*z* = −0.96, *p* = .336, β = −0.44]. Lastly, the proportion of correctly recalled instructions in response to the follow‐up question was 0.73. This indicated that the PM task instruction was correctly encoded in the majority of KS patients, unlike in Experiment 1.

**Table 3:** Performance (number of correct responses) for the prospective memory (PM) task as a function of condition (no‐EFT and EFT) and order in Korsakoff’s syndrome patients and the control group

	Control group	Korsakoff patients			
		no‐EFT		EFT	
	no‐EFT	Order 1^a^	Order 2^a^	Order 1^a^	Order 2^a^
Accuracy (raw score, maximum = 7)					
Mean	6.41	1.0	1.38	2.15	1.92
Median	7	0	0	0	0
Range	7	7	7	7	7
Mode	7	0	0	0	0
Proportion of patients with maximum score	.92	.08	.17	.33	.25

Note.

PM = prospective memory, EFT = episodic future thinking.

^a^ Order 1: no‐EFT then EFT, Order 2: EFT then no‐EFT.

#### Individual differences in the effect of EFT on prospective memory task: Korsakoff’s syndrome patients

**Figure 2:**
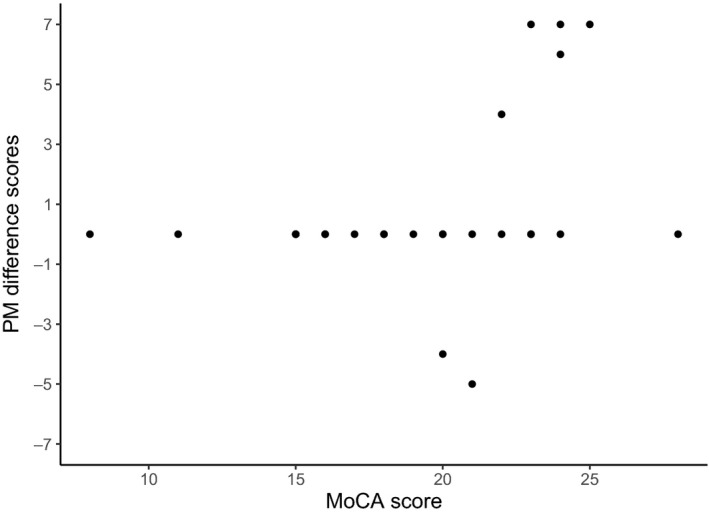
Korsakoff’s syndrome patients difference scores in prospective memory performance between the no‐EFT and EFT condition and individual MoCA scores.

In order to determine whether the significant effect of Instruction (no‐EFT, EFT) on PM performance was explained by individual differences in levels of cognitive functioning, we added MoCA scores as an interaction term in the GLMM. Interestingly, estimates obtained after fitting this model revealed a significant interaction between Instruction and MoCA scores in predicting PM performance [*z* = 3.47, *p* < .001, β = 0.76]. Specifically, as MoCA scores increased, EFT became increasingly effective. Together, these results suggest that the beneficial effects of the EFT instruction on PM performance are present predominantly in high‐functioning KS patients. This finding is visualized in Figure 2.

## General discussion

The present study investigated the extent to which KS patients suffer PM deficits and, since PM is closely linked with independent living, explored whether EFT can be applied as a strategy to boost PM performance. In line with our expectations and previous research (Altgassen *et al.*, 2016; Brunfaut *et al.*, 2000), in two separate experiments, we observed that KS patients performed at floor level on the PM task (Experiment 1) and substantially poorer when compared to a control group of patients with AUD (Experiment 2). In Experiment 1, KS patients were unable to perform the PM task in both the EFT and no‐EFT conditions. The addition of an observational learning component (i.e., instructional video) in Experiment 2 meant that KS patients' PM performance improved compared to Experiment 1. In Experiment 2, where the observational learning component was included, we found that EFT instructions improved PM performance in KS patients. In an additional analysis, we took into account individual differences in cognitive functioning and found that the beneficiary effect of EFT on PM performance was predominantly driven by high‐functioning KS patients. This study is the first to report that EFT may be an effective training strategy for a sub‐group of higher functioning KS patients.

Patients with relatively more intact cognitive functioning based on their MoCA score, benefit significantly more from EFT instructions than those with more impaired cognitive functioning. According to previous research on EFT in Parkinson’s disease (de Vito *et al.*, 2012), the processes involved in simulating a future event place greater demands on executive functions. De Vito *et al. *(2012) found that patients with more cognitive problems due to Parkinson’s disease showed severe impairments in their ability to imagine the future, concluding that executive functions strongly underlie EFT through monitoring and combining different details to form an event. In the light of these findings, KS patients with severe cognitive impairments may suffer the same fate and are unable to carry out such a cognitively complex strategy successfully. Our findings give promise to the idea that the added value of EFT in KS patients is contingent upon the cognitive functioning of that individual.

Interestingly, the results of Experiment 2 were vastly different to those in Experiment 1. In Experiment 1, PM performance in both conditions did not significantly differ from zero, indicating that patients were unable to carry out the task. This was partly due to the patients' inability to successfully encode the PM assignment. The recall rate in Experiment 2 (73.1%) was numerically higher than Experiment 1 (16.6%), indicating that a greater number of patients could recall the PM task instructions following the task in Experiment 2. Correct responses to this question indicate that the patients carried out successful intention formation, the first phase of the PM process. Successful intention formation is necessary to achieve PM (Ellis, 1996; Kliegel, Martin, McDaniel, & Einstein, 2002). We cannot rule out the possibility that this increase in intention formation, and in some patients PM performance, could stem from differences in individual patients (i.e., different patient groups took part in Experiment 1 and 2); however, we believe that it is likely to be explained by the observational learning component (i.e., instructional video concretely displaying the PM task). The importance of visual images in memory functioning is well established, particularly in the consolidation and retention of autobiographical memories (Brewer, Zhao, Desmond, Glover, & Gabrieli, 1998; Greenberg & Rubin, 2003). Studies have implemented this strategy in patient groups' daily life through use of a 'SenseCam', a device which keeps a digital record of the events that a person experiences throughout their day as an external strategy to improve PM. Importantly, these images when seen later by amnesic patients (Hodges et al., 2006) and a KS patient (Svanberg & Evans, 2014) provided powerful triggers for better autobiographical memory. Given the improvement in cued recall (i.e., follow‐up question) in Experiment 2, it is possible that KS patients with highly compromised cognitive functioning could benefit from observing the PM actions in advance by means of concrete visualized examples (i.e., video). According to our results, it is unlikely that an observational learning strategy alone improves PM performance, since we observed that overall in Experiment 2, KS patients still performed poorly when observational learning was included without the EFT instruction. We do, however, show that observational learning has an additional learning effect (on top of EFT) on PM performance, thereby optimizing learning in KS patients. Since we did not directly test the effect of the observational learning manipulation on PM performance in the same group of KS patients, the effectiveness of observational learning on PM performance needs to be more carefully addressed in a future study.

A number of different strategies have been tested with regard to rehabilitating PM in amnesic patients. Recent progress was made using a habitual PM task. Providing immediate feedback for PM failures was shown to be effective for severely amnesic patients in achieving PM tasks both immediately and after 24 hr (Meier *et al.*, 2019). Some strategies have been developed to overcome difficulties in the retrieval and execution of a task; both are later phases of the PM process. For example, two case reports found that external memory aids (Google calendar and a smartwatch) improved PM performance in a patient with Alzheimer’s disease (El Haj, Gallouj, & Antoine, 2017) and a KS patient (Lloyd *et al.*, 2019), respectively. In these studies, patients were prompted for future activities by a reminder message or pop‐up screen on a smartwatch. In order to keep PM performance improved, the patient had to use the external memory aid infinitely. Future thinking, however, stimulates the first stage of the PM process, during intention encoding. Two ways have been proposed in which simulating a future intention exerts a beneficial effect on carrying out the intention at a later point in time, namely via deeper encoding of the intention (i.e., stronger memory traces; Brewer & Marsh, 2010), or by enhancing the association between the context of the ongoing task and the PM cue (Paraskevaides *et al.*, 2010b). Although one could argue that in the present paradigm, both deeper encoding of an intention and enhancing the association between an ongoing context and a PM cue lead to better task performance, it is likely that the event‐based nature of the task leads to a stronger cue–context association. Irrespective of the proposed theoretical mechanism behind better task performance, in the current study, combining an observational learning component and EFT was more beneficial than EFT alone, suggesting a novel therapeutic target in treating PM issues in KS. These results are not only interesting for KS patients, but could be considered as PM rehabilitation strategies in other high‐functioning amnesic patient groups, thereby serving as a promising opportunity for future research.

Taken together, this study illustrates two strategies that may help overcome PM deficits in KS patients. First, an observational learning strategy, where the learner is presented with a visual example of the PM task (e.g., a video of a person turning off a gas cooker after finishing cooking). In our study, observational learning enhanced encoding capacities in this population and thereby led to successful intention formation of the PM task instruction. Second, in a sub‐set of KS patients, pre‐experiencing the specific context of the PM intention through imagination enhanced the retention, initiation and execution of the PM intention. Rehabilitating PM in KS in timely and important, and the current findings have implications for clinical rehabilitation in KS patients. Representing intentions by means of concrete visualized examples as well as EFT training are both simple and cost‐effective strategies that, after further research and development, could be applied in clinical settings to benefit PM. The patients most likely to benefit from EFT training can be selected through an assessment of cognitive functioning. By enhancing PM, these patients may eventually be able to regulate parts of their own life, for example by remembering to take their medication, or to turn off electrical appliances, thereby promoting their seriously compromised autonomy.

## Conflicts of interest

All authors declare no conflict of interest.

## Author contributions

Beth Lloyd (Conceptualization; Data curation; Formal analysis; Methodology; Writing – original draft; Writing – review & editing) Erik Oudman (Conceptualization; Investigation; Methodology; Project administration; Resources; Supervision; Writing – original draft; Writing – review & editing) Mareike Altgassen (Conceptualization; Methodology; Resources; Writing – review & editing) Serge J. W. Walvoort (Project administration; Resources; Writing – review & editing) Roy P. C. Kessels (Resources; Writing – review & editing) Albert Postma (Conceptualization; Investigation; Methodology; Resources; Writing – original draft; Writing – review & editing).

## Data Availability

Lloyd, B. (Utrecht University) (2018): Episodic future thinking in Korsakoff Syndrome patients. DANS. https://doi.org/10.17026/dans‐xek‐5h8y

## References

[bjc12251-bib-0001] Altgassen, M. , Ariese, L. , Wester, A. J. , & Kessels, R. P. C. (2016). Salient cues improve prospective remembering in Korsakoff’s syndrome. British Journal of Clinical Psychology, 55, 123–136. 10.1111/bjc.12099 26577704

[bjc12251-bib-0002] Altgassen, M. , Rendell, P. G. , Bernhard, A. , Henry, J. D. , Bailey, P. E. , Phillips, L. H. , & Kliegel, M. (2015). Future thinking improves prospective memory performance and plan enactment in older adults. Quarterly Journal of Experimental Psychology, 68, 192–204. 10.1080/17470218.2014.956127 25191929

[bjc12251-bib-0003] Alvarez, J. A. , & Emory, E. (2006). Executive function and the frontal lobes: A meta‐analytic review. Neuropsychology Review, 16, 7–42. 10.1007/s11065-006-9002-x 16794878

[bjc12251-bib-0005] Arts, N. J. M. , Walvoort, S. J. W. , & Kessels, R. P. C. (2017). Korsakoff’s syndrome: A critical review. Neuropsychiatric Disease and Treatment, 13, 2875–2890. 10.2147/NDT.S130078 29225466 PMC5708199

[bjc12251-bib-0006] Atance, C. M. , & O’Neill, D. K. (2001). Episodic future thinking. Trends in Cognitive Sciences, 5, 533–539. 10.1016/S1364-6613(00)01804-0 11728911

[bjc12251-bib-0007] Bates, D. , Mächler, M. , Bolker, B. M. , & Walker, S. C. (2015). Fitting linear mixed‐effects models using lme4. Journal of Statistical Software, 67, 1–48. 10.18637/jss.v067.i01

[bjc12251-bib-0008] Brand, M. , Fujiwara, E. , Borsutzky, S. , Kalbe, E. , Kessler, J. , & Markowitsch, H. J. (2005). Decision‐making deficits of Korsakoff patients in a new gambling task with explicit rules: Associations with executive functions. Neuropsychology, 19, 267–277. 10.1037/0894-4105.19.3.267 15910113

[bjc12251-bib-0009] Brand, M. , Fujiwara, E. , Kalbe, E. , Steingass, H. P. , Kessler, J. , & Markowitsch, H. J. (2003). Cognitive estimation and affective judgments in alcoholic Korsakoff patients. Journal of Clinical and Experimental Neuropsychology, 25, 324–334. 10.1076/jcen.25.3.324.13802 12916646

[bjc12251-bib-0010] Brewer, G. A. , & Marsh, R. L. (2010). On the role of episodic future simulation in encoding of prospective memories. Cognitive Neuroscience, 1, 81–88. 10.1080/17588920903373960 24168273

[bjc12251-bib-0011] Brewer, J. B. , Zhao, Z. , Desmond, J. E. , Glover, G. H. , & Gabrieli, J. D. E. (1998). Making memories: Brain activity that predicts how well visual experience will be remembered. Science, 281, 1185–1187. 10.1126/science.281.5380.1185 9712581

[bjc12251-bib-0012] Brion, M. , Pitel, A. L. , Beaunieux, H. , & Maurage, P. (2014). Revisiting the continuum hypothesis: Toward an in‐depth exploration of executive functions in Korsakoff syndrome. Frontiers in Human Neuroscience, 8, 498 10.3389/fnhum.2014.00498 25071526 PMC4081760

[bjc12251-bib-0013] Brunfaut, E. , Vanoverberghe, V. , & D’Ydewalle, G. (2000). Prospective remembering of Korsakoffs and alcoholics as a function of the prospective‐memory and on‐going tasks. Neuropsychologia, 38, 975–984. 10.1016/S0028-3932(00)00016-6 10775708

[bjc12251-bib-0014] Butters, N. , Tarlow, S. , Cermak, L. S. , & Sax, D. (1976). A comparison of the information processing deficits of patients with huntington’s chorea and Korsakoff’s Syndrome. Cortex, 12, 134–144. 10.1016/S0010-9452(76)80017-2 133786

[bjc12251-bib-0015] de Vito, S. , Gamboz, N. , Brandimonte, M. A. , Barone, P. , Amboni, M. , & Della Sala, S. (2012). Future thinking in Parkinson’s disease: An executive function? Neuropsychologia, 50, 1494–1501. 10.1016/j.neuropsychologia.2012.03.001 22406693

[bjc12251-bib-0016] Einstein, G. O. , & McDaniel, M. A. (1990). Normal aging and prospective memory. Journal of Experimental Psychology: Learning, Memory, and Cognition, 16, 717–726. 10.1037/0278-7393.16.4.717 2142956

[bjc12251-bib-0017] El Haj, M. , Gallouj, K. , & Antoine, P. (2017). Google calendar enhances prospective memory in Alzheimer’s Disease: A case report. Journal of Alzheimer’s Disease, 57, 285–291. 10.3233/JAD-161283 28222535

[bjc12251-bib-0018] Ellis, J. (1996). Prospective memory or the realisation of delayed intentions: A conceptual framework for research In Brandimonte M., Einstein G. O. & McDaniel M. A. (Eds.), Prospective memory: Theory and applications (pp. 1–22). Hillsdale, NJ: Erlbaum.

[bjc12251-bib-0019] Fama, R. , Marsh, L. , & Sullivan, E. V. (2004). Dissociation of remote and anterograde memory impairment and neural correlates in alcoholic Korsakoff sydrome. Journal of the International Neuropsychological Society, 10, 427–441. 10.1017/S135561770410310X 15147600

[bjc12251-bib-0020] Fama, R. , Pitel, A. L. , & Sullivan, E. V. (2012). Anterograde episodic memory in Korsakoff syndrome. Neuropsychology Review, 22, 93–104. 10.1007/s11065-012-9207-0 22644546 PMC4724416

[bjc12251-bib-0021] Fujiwara, E. , Brand, M. , Borsutzky, S. , Steingass, H. P. , & Markowitsch, H. J. (2008). Cognitive performance of detoxified alcoholic Korsakoff syndrome patients remains stable over two years. Journal of Clinical and Experimental Neuropsychology, 30, 576–587. 10.1080/13803390701557271 17852615

[bjc12251-bib-0022] Gollwitzer, P. M. (1999). Implementation intentions: Strong effects of simple plans. American Psychologist, 54, 493–503. 10.1037/0003-066X.54.7.493

[bjc12251-bib-0023] Greenberg, D. L. , & Rubin, D. C. (2003). The neuropsychology of autobiographical memory. Cortex, 39, 687–728. 10.1016/S0010-9452(08)70860-8 14584549

[bjc12251-bib-0024] Hodges, S. , Williams, L. , Berry, E. , Izadi, S. , Srinivasan, J. , Butler, A. , … Wood, K. (2006). SenseCam: A retrospective memory aid. Lecture Notes in Computer Science, 10.1007/11853565_11

[bjc12251-bib-0025] Joyce, E. M. , & Robbins, T. W. (1991). Frontal lobe function in Korsakoff and non‐Korsakoff alcoholics: Planning and spatial working memory. Neuropsychologia, 29, 709–723. 10.1016/0028-3932(91)90067-I 1944873

[bjc12251-bib-0026] Kessels, R. P. C. , Postma, A. , Wester, A. J. , & De Haan, E. H. F. (2000). Memory for object locations in Korsakoff’s amnesia. Cortex, 36, 47–57. 10.1016/S0010-9452(08)70835-9 10728896

[bjc12251-bib-0027] Kliegel, M. , Martin, M. , McDaniel, M. A. , & Einstein, G. O. (2002). Complex prospective memory and executive control of working memory: A process model. Psychological Test and Assessment Modeling, 44, 303–318.

[bjc12251-bib-0028] Kopelman, M. D. (2002). Disorders of memory. Brain, 125, 2152–2190. 10.1093/brain/awf229 12244076

[bjc12251-bib-0029] Leitz, J. R. , Morgan, C. J. A. , Bisby, J. A. , Rendell, P. G. , & Curran, H. V. (2009). Global impairment of prospective memory following acute alcohol. Psychopharmacology (Berl), 205, 379–387. 10.1007/s00213-009-1546-z 19440700

[bjc12251-bib-0030] Lloyd, B. , Oudman, E. , Altgassen, M. , & Postma, A. (2019). Smartwatch aids time‐based prospective memory in Korsakoff syndrome: A case study. Neurocase, 25, 21–25. 10.1080/13554794.2019.1602145 30966873

[bjc12251-bib-0031] Meier, B. , Fanger, S. , Toller, G. , Matter, S. , Müri, R. , & Gutbrod, K. (2019). Amnesic patients have residual prospective memory capacities. Clinical Neuropsychologist, 33, 606–621. 10.1080/13854046.2018.1438516 29436258

[bjc12251-bib-0032] Mioni, G. , Bertucci, E. , Rosato, A. , Terrett, G. , Rendell, P. G. , Zamuner, M. , & Stablum, F. (2017). Improving prospective memory performance with future event simulation in traumatic brain injury patients. British Journal of Clinical Psychology, 56, 130–148. 10.1111/bjc.12126 28093771

[bjc12251-bib-0033] Miyake, A. , Friedman, N. P. , Emerson, M. J. , Witzki, A. H. , Howerter, A. , & Wager, T. D. (2000). The unity and diversity of executive functions and their contributions to complex. Cognitive Psychology, 41, 49–100. 10.1006/cogp.1999.0734 10945922

[bjc12251-bib-0034] Moerman‐Van Den Brink, W. G. , Van Aken, L. , Verschuur, E. M. L. , Walvoort, S. J. W. , Egger, J. I. M. , & Kessels, R. P. C. (2019). Executive dysfunction in patients with Korsakoff’s syndrome: A theory‐driven approach. Alcohol and Alcoholism, 54, 23–29. 10.1093/alcalc/agy078 30407502

[bjc12251-bib-0035] Nasreddine, Z. S. , Phillips, N. A. , Bédirian, V. , Charbonneau, S. , Whitehead, V. , Collin, I. , … Chertkow, H. (2005). The Montreal Cognitive Assessment, MoCA: A brief screening tool for mild cognitive impairment. Journal of the American Geriatrics Society, 53, 695–699. 10.1111/j.1532-5415.2005.53221.x 15817019

[bjc12251-bib-0004] Nuckols, C. C. , Nuckols, C. C. (2013). The diagnostic and statistical manual of Mental Disorders, (DSM‐5). Philadelphia: American Psychiatric Association.

[bjc12251-bib-0036] Paraskevaides, T. , Morgan, C. J. A. , Leitz, J. R. , Bisby, J. A. , Rendell, P. G. , & Curran, H. V. (2010a). Drinking and future thinking: Acute effects of alcohol on prospective memory and future simulation. Psychopharmacology (Berl), 208, 301–308. 10.1007/s00213-009-1731-0 19967530

[bjc12251-bib-0037] Peirce, J. W. (2007). PsychoPy‐psychophysics software in Python. Journal of Neuroscience Methods, 162, 8–13. 10.1016/j.jneumeth.2006.11.017 17254636 PMC2018741

[bjc12251-bib-0038] Pitel, A. L. , Beaunieux, H. , Witkowski, T. , Vabret, F. , De La Sayette, V. , Viader, F. , … Eustache, F. (2008). Episodic and working memory deficits in alcoholic korsakoff patients: The continuity theory revisited. Alcoholism: Clinical and Experimental Research, 32, 1229–1241. 10.1111/j.1530-0277.2008.00677.x 18482159

[bjc12251-bib-0039] Postma, A. , Morel, S. G. , Slot, M. E. , Oudman, E. , & Kessels, R. P. C. (2018). Forgetting the new locations of one’s keys: Spatial‐memory interference in Korsakoff’s amnesia. Experimental Brain Research, 236, 1861–1868. 10.1007/s00221-018-5266-7 29680910 PMC6010480

[bjc12251-bib-0040] Postma, A. , Van Asselen, M. , Keuper, O. , Wester, A. J. , & Kessels, R. P. C. (2006). Spatial and temporal order memory in Korsakoff patients. Journal of the International Neuropsychological Society, 12, 327–336. 10.1017/S1355617706060449 16903125

[bjc12251-bib-0041] Prigatano, G. P. , & Klonoff, P. S. (1998). A clinician’s rating scale for evaluating impaired self‐awareness and denial of disability after brain injury. Clinical Neuropsychologist, 12, 56–67. 10.1076/clin.12.1.56.1721

[bjc12251-bib-0042] Schacter, D. L. , & Addis, D. R. (2007). Constructive memory: The ghosts of past and future. Nature, 445, 27–27. 10.1038/445027a 17203045

[bjc12251-bib-0043] Schacter, D. L. , Addis, D. R. , & Buckner, R. L. (2007). Remembering the past to imagine the future: The prospective brain. Nature Reviews Neuroscience, 8, 657–661. 10.1038/nrn2213 17700624

[bjc12251-bib-0044] Shohamy, D. , Myers, C. E. , Grossman, S. , Sage, J. , Gluck, M. A. , & Poldrack, R. A. (2004). Cortico‐striatal contributions to feedback‐based learning: Converging data from neuroimaging and neuropsychology. Brain, 127, 851–859. 10.1093/brain/awh100 15013954

[bjc12251-bib-0045] Svanberg, J. , & Evans, J. J. (2014). Impact of SenseCam on memory, identity and mood in Korsakoff’s syndrome: A single case experimental design study. Neuropsychological Rehabilitation, 24, 400–418. 10.1080/09602011.2013.814573 24131241

[bjc12251-bib-0046] Tulving, E. (1983). Elements of episodic memory. Canadian Psychology, 26, 235–238.

[bjc12251-bib-0047] Van Tilborg, I. A. D. A. , Kessels, R. P. C. , & Hulstijn, W. (2011). Learning by observation and guidance in patients with Alzheimer’s dementia. NeuroRehabilitation, 29, 295–304. 10.3233/NRE-2011-0705 22142763

[bjc12251-bib-0048] Verhage, F. (1964). Intelligentie en leeftijd: onderzoek bij Nederlanders van twaalf tot zevenzeventig jaar [Intelligence and Age: study with Dutch people aged 12 to 77]. Assen: Van Gorcum.

